# Recommendation Regarding Universal Motorcycle Helmet Laws — Community Preventive Services Task Force

**Published:** 2014-06-27

The Community Preventive Services Task Force recently posted new information on its website: “Use of Motorcycle Helmets: Universal Helmet Laws.” The task force recommends universal motorcycle helmet laws (laws that apply to all motorcycle operators and passengers) based on strong evidence of effectiveness. Evidence indicates that universal helmet laws increase helmet use, decrease motorcycle-related fatal and nonfatal injuries, and are substantially more effective than no law or only partial motorcycle helmet laws. This information is available at http://www.thecommunityguide.org/mvoi/motorcyclehelmets/helmetlaws.html.

Established in 1996 by the U.S. Department of Health and Human Services, the task force is an independent, nonfederal, uncompensated panel of public health and prevention experts whose members are appointed by the Director of CDC. The task force provides information for a wide range of decision makers on programs, services, and policies aimed at improving population health. Although CDC provides administrative, research, and technical support for the task force, the recommendations developed are those of the task force and do not undergo review or approval by CDC.

